# Dihydroisocoumarins from the Mangrove-Derived Fungus *Penicillium citrinum*

**DOI:** 10.3390/md14100177

**Published:** 2016-10-10

**Authors:** Guo-Lei Huang, Xue-Ming Zhou, Meng Bai, Yu-Xin Liu, Yan-Lei Zhao, You-Ping Luo, Yan-Yan Niu, Cai-Juan Zheng, Guang-Ying Chen

**Affiliations:** Key Laboratory of Tropical Medicinal Plant Chemistry of Ministry of Education, College of Chemistry and Chemical Engineering, Hainan Normal University, Haikou 571158, Hainan, China; huangguolei1982@hainnu.edu.cn (G.-L.H.); xueming2009211@126.com (X.-M.Z.); xxbai2014@163.com (M.B.); 18174202788@163.com (Y.-X.L.); 18689506770@163.com (Y.-L.Z.); luoyouping@gmail.com (Y.-P.L.); niuyanyan1986@126.com (Y.-Y.N.)

**Keywords:** *Penicillium citrinum*, metabolites, dihydroisocoumarin, antibacterial activity, cytotoxic activity

## Abstract

Three new dihydroisocoumarin penicimarins G–I (**1**–**3**), together with one known dihydroisocoumarin (**4**) and three known meroterpenoids (**5**–**7**), were obtained from a fungus *Penicillium citrinum* isolated from the mangrove *Bruguiera sexangula* var. *rhynchopetala* collected in the South China Sea. Their structures were elucidated by the detailed analysis of spectroscopic data. The absolute configuration of **1** was determined by the X-ray diffraction analysis using Cu Kα radiation. The absolute configurations of **2** and **3** were determined by comparison of their circular dichroism (CD) spectra with the literature. All compounds were evaluated for their antibacterial activities and cytotoxic activities.

## 1. Introduction

**Figure 1:**
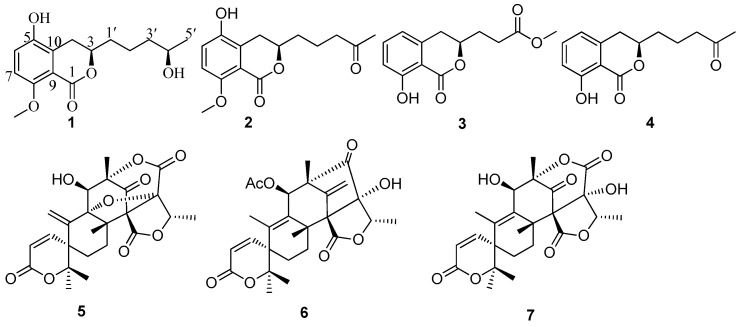
Structures of compounds **1**–**7**.

Marine-derived fungi have attracted increasing attention from those seeking new pharmaceutically useful natural products in recent years [1]. In particular, secondary metabolites isolated from the marine-derived fungi in the genus *Penicillium* can produce various bioactive metabolites, such as antifouling polyketides [2], antibacterial communal A [3], cytotoxic penicinoline [4], antioxidant terrestrols [5], and antileukemic sorbicillactone A [6]. These results have drawn the attention of both pharmaceutical and natural product chemists. In our search for new bioactive natural products from marine fungi in the South China Sea, we have found several bioactive compounds, including anthraquinone derivatives, cytochalasins, lumazine peptides, benzopyrans, stemphol sulfates, triterpenes, and *α*-pyrone derivatives [7,8,9,10,11,12,13]. Our previous investigation on the endophytic fungus *Penicillium citrinum* has resulted in the discovery of one new benzopyran derivative (2R*,4R*)-3,4-dihydro-5-methoxy-2-methyl-2H-1-benzopyran-4-ol [10]. Further, chemical investigation of the fermentation broth of the *Penicillium citrinum* resulted in the isolation of three new dihydroisocoumarin penicimarins (G–I (**1**–**3**)), together with one known dihydroisocoumarin (aspergillumarin A (**4**)) [14], and three known meroterpenoids (dehydroaustin (**5**) [15], 11β-acetoxyisoaustinone (**6**) [15], and austinol (**7**) [16]) (Figure 1). All isolated metabolites (**1**–**7**) were evaluated for their antibacterial and cytotoxic activities. Herein we report the isolation, structure elucidation, and biological activities of these compounds.

## 2. Results and Discussion

**Figure 2:**
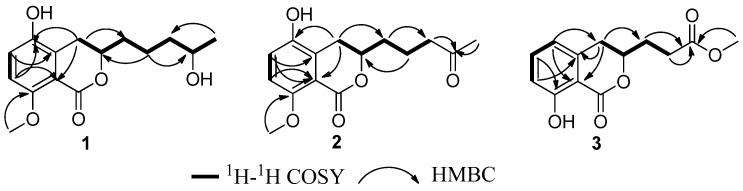
^1^H–^1^H COSY correlations and key HMBC correlations of Compounds **1**–**3**.

**Figure 3:**
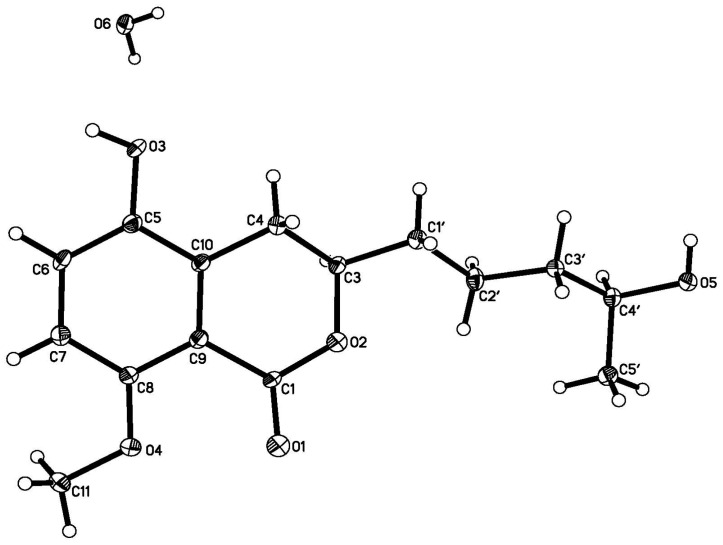
X-ray structure of Compound **1**.

**Figure 4:**
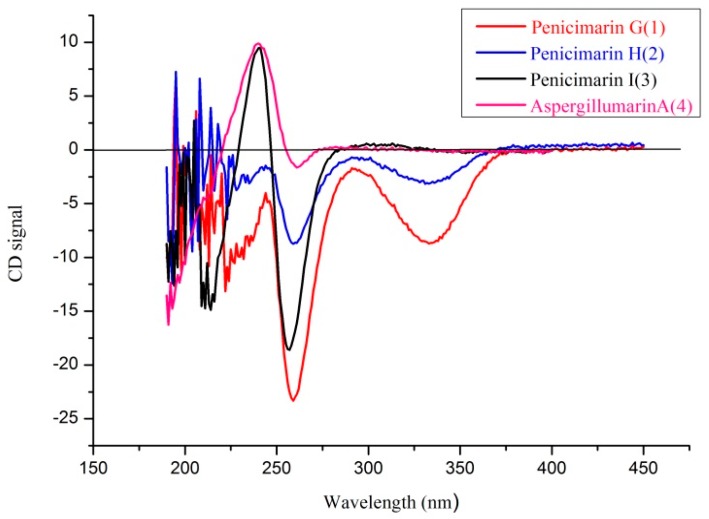
Circular dichroism (CD) spectra of Compounds **1**–**4**.

Compound **1** was obtained as colorless crystals. Its molecular formula of C_15_H_20_O_5_ (six degrees of unsaturation) was determined by HR-ESI–MS and combined with ^1^H and ^13^C NMR spectroscopic data. In the ^1^H NMR spectrum (Table 1), the signals and the coupling constants at δ_H_ 7.05 (d, *J* = 8.4 Hz) and 6.90 (d, *J* = 8.4 Hz) indicated the presence of a 1,2,3,4-tetrasubstituted benzene system. Two oxygenated methine proton signals at δ_H_ 4.39 (m) and 3.75 (m), one methoxyl group at δ_H_ 3.82 (s), one methyl group at δ_H_ 1.17 (d, *J* = 6.2 Hz), and four methylene groups at δ_H_ (3.16 (dd, *J* = 16.8, 3.0 Hz) and 2.58 (dd, *J* = 16.8, 11.6 Hz)), (1.84 (m) and 1.70 (m)), 1.59 (m) and 1.55 (m) were also observed. The ^1^H NMR and ^13^C NMR spectra indicated that this compound included four methylenes, two methines, one methoxy, and one methyl. The ^13^C NMR spectrum revealed the signals for six aromatic carbons and one carbonyl carbon, including one lactone carbonyl carbon at δ_C_ 165.8. Thus, these spectroscopic features suggested that **1** was very similar to penicimarin B [17]. The obvious difference in the ^1^H NMR spectrum was the absence of one aromatic proton signal at δ_H_ 6.79 (d, *J* = 7.2 Hz, H-5) in **1**. Furthermore, in the ^13^C NMR spectra, the C-5 signal moved downfield significantly [δ_C_ 148.2 (C) in **1** vs 119.3 (CH)] in penicimarin B, indicating an aromatic proton is replaced by a hydroxyl group. The ^1^H,^1^H-COSY, HMQC, and HMBC spectra allowed the complete assignment for **1** (Figure 2). The relative configuration of **1** could, however, not be confirmed unambiguously. Crystallization of **1** from MeOH afforded colorless crystals, which gave an X-ray crystal structure with a Flack parameter of 0.0 (3) (Figure 3). The absolute configuration of C-3 was also determined by circular dichroism (CD) spectroscopy (Figure 4). The negative circular dichroism at 259 nm (Figure 4) suggested the *R* configuration at C-3, by comparison with data for dihydroisocoumarins described in the literature [18]. Thus, compound **1** was named as penicimarin G, and the absolute configuration of **1** was defined as (3*R*, 4′*R*).

Compound **2** was isolated as colorless crystals with the molecular formula assigned as C_15_H_18_O_5_ (seven degrees of unsaturation) on the basis of its HR-ESI–MS and combined with ^1^H and ^13^C NMR spectroscopic data. The 1D NMR data of **2** was very similar with that of **1**, except for the presence of a carbonyl signal (δ_C_ 211.4) for C-4′ in **2** and the absence of one oxygenated methine proton signal (δ_C_ 68.3) for C-4′ in **1**. This was corroborated by the HMBC correlations from H-3′ and H-5′ to C-4′ (Figure 2). The ^1^H–^1^H-COSY, HMQC, and HMBC spectra allowed the complete assignment for **1**. Thus, compound **2** was named as penicimarin H, and the absolute configuration of C-3 was also determined to be *R* by CD spectra (Figure 4) [18].

Compound **3** was obtained as a colorless oil. Its molecular formula of C_13_H_14_O_5_ (seven degrees of unsaturation) was determined by HR-ESI–MS and combined with ^1^H and ^13^C NMR spectroscopic data. In the ^1^H NMR spectrum (Table 1), the proton signals and the coupling constants at δ_H_ 7.42 (dd, *J* = 8.0, 7.6 Hz), 6.89 (d, *J* = 8.0 Hz), and 6.70 (d, *J* = 7.6 Hz) indicated the presence of an 1,2,3-trisubstituted benzene system. One hydrogen-bonded hydroxyl group at δ_H_ 10.9 (s), one oxygenated methine proton signal at δ_H_ 4.65 (m), one methoxyl group at δ_H_ 3.70 (s), and three methylene groups at δ_H_ 2.92 (m), 2.62 (m), and 2.13 (m) were also observed. The ^1^H NMR and ^13^C NMR spectra included three methylenes, one methine, and one methoxy. The ^13^C NMR data exhibited the presence of six aromatic carbons and two carbonyl carbons, including two lactone carbonyl carbons at δ_C_ 173.2 (C) and 169.7 (C). Thus, these spectroscopic features suggested that **3** was very similar to aspergillumarin A (**4**) [14]. The obvious differences in the ^1^H NMR spectra were the absence of one methylene signal at δ_H_ 1.76 (m), a singlet methyl signal at δ_H_ 2.16 (s), and the presence of a methoxy signal at δ_H_ 3.70 (s) in **3**. The position of this methoxy group was confirmed by the HMBC correlation from 4′-OMe to C-3′ (Figure 1). The ^1^H,^1^H-COSY, HMQC, and HMBC spectra allowed the complete assignment for **3**. Thus, compound **3** was named as penicimarin I, and the absolute configuration of C-3 was also determined to be *R* by CD spectra (Figure 4) [18].

By comparing physical and spectroscopic data with literature values, the four known compounds were identified as aspergillumarin A (**4**) [14] and three meroterpenoids: dehydroaustin (**5**) [15], 11β-acetoxyisoaustinone (**6**) [15], and austinol (**7**) [16]. The absolute configuration of C-3 in compound **4** was determined to be *R* by comparison with the CD spectrum and the optical rotation data of those reported in the literature [14,18].

More detailed spectra of new compounds are available in the Supplementary Materials (Figures S1–S18).

All the isolated compounds were evaluated for their antibacterial activities against five terrestrial pathogenic bacteria and two marine pathogenic bacteria. Compounds **1**, **2**, and **5**–**7** exhibited selective antibacterial activity (Table 2). Compounds **2** and **7** showed moderate activity against *Staphylococcus epidermidis* and *S. aureus* with the same minimum inhibitory concentration (MIC) values of 10 μM. Compounds **1** and **2** showed a broad spectrum of antibacterial activity against the five pathogenic bacteria *S. epidermidis*, *S. aureus*, *Escherichia coli*, *Bacillus cereus*, and *Vibrio alginolyticus.* All compounds were also tested for cytotoxic activity against HeLa, MCF-7, and A549 cells, however, these compounds showed no cytotoxic activity (IC_50_ > 50 μM).

## 3. Materials and Methods

### 3.1. General Experimental Procedures

Optical rotations were measured on a JASCO P-1020 digital polarimeter. IR spectra were recorded on a Thermo Nicolet 6700 (using KBr disks) spectrophotometer (Thermo Scientific, Madison, WI, USA). Both 1D and 2D NMR spectra were measured on a Bruker AV-400 (Bruker Corporation, Switzerland) instrument with TMS as the internal standard. HR-ESI–MS spectra were obtained on the Bruker Daltonics Apex-Ultra 7.0 T (Bruker Corporation, Billerica, MA, USA) and the Q-TOF Ultima Global GAA076 LC mass spectrometer. Single-crystal data: Agilent Gemini Ultra X-ray single crystal diffractometer (Cu Kα radiation). CD spectra were recorded on a MOS-450 spectrometer. Prep. HPLC were used for Agilent 1260 prep-HPLC system with an Agilent C18 analytical HPLC column (4.6 × 250 mm, 5 μm) and semi-preparative column (9.4 × 250 mm, 7 μm). Sephadex LH-20 (Pharmacia Co. Ltd, Sandwich, UK) and Silica gel (200–300 mesh, 300–400 mesh Qingdao Marine Chemical Factory, Qingdao, China) were used for column chromatography (CC). All solvents were purchased from Xilong Chemical Reagent Factory (Guangzhou, China).

### 3.2. Fungal Materials

The fungal strain *Penicillium citrinum* HL-5126 was isolated from the mangrove *Bruguiera sexangula* var. *rhynchopetala* collected in the South China Sea in August, 2012. The strain was deposited in the Key Laboratory of Tropical Medicinal Plant Chemistry of Ministry of Education, College of Chemistry and Chemical Engineering, Hainan Normal University, Haikou, China, with the access code HL-5126. The fungal strain was cultivated in 20 L potato glucose liquid medium (15 g of glucose and 30 g of sea salt in 1 L of potato infusion, in 1 L Erlenmeyer flasks each containing 300 mL of culture broth) at 25 °C without shaking for 4 weeks.

### 3.3. Identification of Fungus

The fungus was identified according to its morphological characteristics and by comparison of the ITS sequence amplification, primer pair ITS1F and ITS4 and sequencing of the internal transcribed spacer (ITS) region. The sequence data has been submitted to GenBank, with an accession number KJ466981, and the fungal strain was identified as *Penicillium citrinum*.

### 3.4. Extraction and Isolation

The fungal cultures were filtered through cheesecloth, and the filtrate was extracted with EtOAc (3 × 20 L, 24 h each). The organic extracts were concentrated in vacuo to yield an oily residue (10.2 g), which was subjected to silica gel column chromatography (CC) (petroleum ether, EtOAc v/v, gradient 100:0–0:100) to generate six fractions (Fr. 1–Fr. 5). Fr. 3 was isolated by CC on silica gel eluted with petroleum ether–EtOAc (v/v, gradient 100:10–10:100), and then subjected to Sephadex LH-20 CC eluting with mixtures of Petroleum ether:CHCl_3_:MeOH (2:1:1), and further purified by using HPLC on an ODS semi-preparative column (Agilent C18, 9.4 × 250 mm, 7 μm, 2 mL/min) eluted with 80% MeOH/H_2_O to obtain compound **1** (5.0 mg). Fr. 4 was isolated by CC on silica gel eluted with petroleum ether–EtOAc (v/v, gradient 100:10–10:100), and then subjected to Sephadex LH-20 CC eluting with mixtures of CHCl_3_–MeOH (1:1) to obtain Fr. 4.2 (400.0 mg), Fr. 4.3 (106.0 mg). Fr. 4.2 was subjected to repeated Sephadex LH-20 CC (CHCl_3_/MeOH, v/v, 1:1) and further purified by using HPLC on an ODS semi-preparative column (Agilent C18, 9.4 × 250 mm, 7 μm, 2 mL/min) eluted with 70% MeOH/H_2_O to obtain compounds **4** (6.0 mg) **5** (8.0 mg), **6** (10.0 mg), and **7** (7.0 mg). Fr. 4.3 was subjected to repeated Sephadex LH-20 CC (MeOH) and further purified by using HPLC on an ODS semi-preparative column (Agilent C18, 9.4 × 250 mm, 7 μm, 2 mL/min) eluted with 60% MeOH/H_2_O to obtain compounds **2** (5.0 mg) and **3** (6.2 mg).

Penicimarin G (**1**): Colorless crystals. ${\left[\alpha \right]}_{D}^{24}$ −29.0 (*c* = 0.25, MeOH). mp 118.8–120.4 °C; *λ*_max_ (log ε) 220 (3.02), 246 (2.50), 312 (1.62) nm; CD (*c* 2 × 10^−4^ mol/L, MeOH) *λ*_max_ (Δε), 259 (−23.0), 280 (−2.5), 330 (−13.6); IR (KBr) *ν*_max_ 3518, 1712, 1602, 1272, 1250, 1230, 1112, 1082, 1064, 798 cm^−1^; ^1^H and ^13^C NMR see Table 1; HRESIMS *m*/*z* 281.1384 [M + H]^+^ (calcd. for C_15_H_21_O_5_, 281.1383).

Penicimarin H (**2**): Colorless crystals. ${\left[\alpha \right]}_{D}^{24}$ −24.6 (*c* = 0.25, MeOH). mp 136.9–137.6 °C. *λ*_max_ (log *ε*) 220 (3.02), 246 (2.50), 312 (1.62) nm; CD (5.2 × 10^−4^ mol/L, MeOH) *λ*_max_ (Δ*ε*) 239 (+2.00), 257 (−8.60), 290 (−1.52), 329 (−3.10); IR (KBr) *ν*_max_ 3518, 1712, 1602, 1272, 1250, 1230, 1112, 1082, 1064, 798 cm^−1^; ^1^H and ^13^C NMR see Table 1; HRESIMS *m*/*z* 579.2200 [2M + Na]^+^ (calcd. for C_30_H_36_O_10_Na, 579.2200).

Penicimarin I (**3**): Colorless crystals. ${\left[\alpha \right]}_{D}^{24}$ −12.0 (*c* = 0.25, MeOH). *λ*_max_ (log ε) 220 (3.02), 246 (2.50), 312 (1.62) nm; CD (4.0 × 10^−4^ mol/L, MeOH) *λ*_max_ (Δε) 240 (+9.60), 260 (−2.65), 328 (−0.16); IR (KBr) *ν*_max_ 3518, 1712, 1602, 1272, 1250, 1230, 1112, 1082, 1064, 798 cm^−1^; ^1^H and ^13^C NMR see Table 1; HRESIMS *m*/*z* 273.0733 [M + Na]^+^ (calcd. for C_13_H_14_O_5_Na, 273.0740).

X-ray Crystallographic Analysis of **1**. Colorless crystals of **1** were obtained from MeOH. Single-crystal X-ray diffraction data were collected on a *Xcalibur*, *Atlas*, *Gemini ultra* diffractometer with Cu K*α* radiation (λ = 1.54180 Å) at 120.01(10) K, respectively. The structure was solved by direct methods (ShelXS) and refined with the ShelXL refinement package using least squares minimization. All non-hydrogen atoms were refined anisotropically, and all hydrogen atoms were placed in idealized positions and refined relatively isotropically with a riding model. Crystallographic data for **1** has been deposited in the Cambridge Crystallographic Data Centre with the deposition number CCDC 1489691, respectively. Copies of the data can be obtained, free of charge, on application to the Director, CCDC, 12 Union Road, Cambridge CB21EZ, UK (Fax: +44-(0)1223-336033, or e-mail: deposit@ccdc.cam.ac.uk).

Crystal data for **1**: C_15_H_22_O_6_·H_2_O, *M*_r_ = 298.33, orthorhombic, *a* = 8.1247(2) Å, *b* = 11.8703(4) Å, *c* = 15.5875(5) Å, α = 90°, β = 90°, γ = 90°, *V* = 1503.30(8) Å^3^, space group *P*2_1_2_1_2_1_, *Z* = 4, *D*_x_ = 1.318 mg/mm^3^, *μ* (Cu K*α*) = 0.848 mm^−1^, and *F*(000) = 640. Independent reflections: 2681 (*R*_int_ = 0.083). The final *R*_1_ values were 0.0389, *wR*_2_ = 0.0979 (*I* > 2σ(*I*)). Flack parameter = 0.0(3).

### 3.5. Biological Assays

Antibacterial activity was determined against five terrestrial pathogenic bacteria, including *Staphylococcus epidermidis*, *S. aureus*, *Escherichia coli*, *Pseudomonas aeruginosa* and *Bacillus cereus*, and two marine pathogenic bacteria *Vibrio parahaemolyticus* and *V. alginolyticus* by the microtitre plate assay method [19]. Ciprofloxacin was used as the positive control. Cytotoxic activities of all compounds against HeLa, MCF-7 and A549 cell lines were evaluated by the MTT method [20]. Epirubicin was used as a positive control.

## 4. Conclusions

Seven secondary metabolites, including three new dihydroisocoumarin penicimarins G–I (**1**–**3**), together with one known dihydroisocoumarin (**4**) and three known meroterpenoids (**5**–**7**), were obtained from a fungus *P. citrinum* isolated from the mangrove *B. sexangula* var. *rhynchopetala* collected in the South China Sea. Their structures were elucidated by the detailed analysis of spectroscopic data. The absolute configuration of **1** was determined by the X-ray diffraction analysis using Cu Kα radiation. The absolute configurations of **2**–**3** were determined by comparison of their CD spectra with literature. All compounds were evaluated for their antibacterial activities and cytotoxic activities.

## Figures and Tables

**Table 1:** NMR spectroscopic data (400/100 MHz) for Compounds **1**–**3**.

Position	1 *^a^*		2 *^a^*		3 *^b^*	
	δ_C_, Type	δ_H_ (*J* in Hz)	δ_C_, Type	δ_H_ (*J* in Hz)	δ_C_, Type	δ_H_ (*J* in Hz)
1	165.8, C		165.7, C		169.5, C	
3	79.6, CH	4.39 (m)	79.4, CH	4.39 (m)	78.4, CH	4.65 (m)
4	28.7, CH_2_	3.16 (dd, 16.8, 3.0) 2.58 (dd, 16.8, 11.6)	28.6, CH_2_	3.15 (dd, 16.8, 3.0) 2.59 (dd, 16.8, 12.0)	32.9, CH_2_	2.92 (m)
5	148.2, C		148.2, C		118.0, CH	6.89 (d, 8.0)
6	122.3, CH	7.05 (d, 8.4)	122.3, CH	7.05 (d, 9.0)	136.2, CH	7.42 (dd, 8.0, 7.6)
7	112.8, CH	6.90 (d, 8.4)	112.8, CH	6.90 (d, 9.0)	116.3, CH	6.70 (d, 7.6)
8	155.8, C		155.8, C		162.2, C	
9	114.8, C		114.7, C		139.0, C	
10	129.5, C		129.4, C		108.3, C	
1′	35.8, CH_2_	1.84 (m), 1.70 (m)	35.1, CH_2_	1.79 (m)	29.8, CH_2_	2.13 (m)
2′	23.5, CH_2_	1.59 (m)	20.3, CH_2_	1.72 (m)	29.2, CH_2_	2.62 (dd, 14.4, 7.2)
3′	39.8, CH_2_	1.55 (m)	43.6, CH_2_	2.58 (m)	173.2	
4′	68.3, CH	3.75 (m)	211.4, CH		51.8, CH_3_	3.70 (s)
5′	22.3, CH_3_	1.17 (d, 6.2)	29.8, CH_3_	2.15 (s)		
8-OMe	56.7, CH_3_	3.82 (s)	56.7, CH_3_	3.81 (s)		
8-OH						10.94 (s)

*^a^* Recorded in Methanol-*d*_4_; *^b^* Recorded in CDCl_3_.

**Table 2:** Antibacterial activity for Compounds **1**, **2** and **5**–**7**.

Compound	Minimum Inhibitory Concentration (MIC, μM)				
	*S. epidermidis*	*S. aureus*	*E. coli*	*B. cereus*	*V. alginolyticus*
**1**	20	20	20	20	20
**2**	10	20	>20	20	20
**5**	20	>20	>20	>20	>20
**6**	20	>20	>20	>20	>20
**7**	10	>20	>20	20	>20
Ciprofloxacin *^a^*	0.30	0.30	0.60	1.20	1.25

*^a^* Ciprofloxacin was used as a positive control.

## Supplementary Materials

The following are available online at www.mdpi.com/1660-3397/14/10/177/s1. Figure S1: 1H NMR (400 MHz, MeOD-*d*_4_) spectrum of Compound 1, Figure S2: 13C NMR (100 MHz, MeOD-*d*_4_) spectrum of Compound 1, Figure S3: HMQC (MeOD-*d*_4_) spectrum of Compound 1, Figure S4: 1H–1H COSY (MeOD-*d*_4_) spectrum of Compound 1, Figure S5: HMBC spectrum (MeOD-*d*_4_) of compound 1, Figure S6: HRESIMS spectrum of Compound 1, Figure S7: 1H NMR (400 MHz, MeOD-*d*_4_) spectrum of Compound 2, Figure S8: 13C NMR (100 MHz, MeOD-*d*_4_) spectrum of Compound 2, Figure S9: HMQC (MeOD-*d*_4_) spectrum of Compound 2, Figure S10: 1H–1H COSY (MeOD-*d*_4_) spectrum of Compound 2, Figure S11: HMBC (MeOD-*d*_4_) spectrum of Compound 2, Figure S12: HRESIMS spectrum of Compound 2, Figure S13: 1H NMR (400 MHz, CDCl3) spectrum of Compound 3, Figure S14: 13C NMR (100 MHz, CDCl3) spectrum of Compound 3, Figure S15: HMQC (CDCl3) spectrum of Compound 3, Figure S16: 1H–1H COSY (CDCl3) spectrum of Compound 3, Figure S17: HMBC (CDCl3) spectrum of Compound 3, Figure S18: HRESIMS spectrum of Compound 3.
