# Supplementary material for: Dihydroisocoumarins from the Mangrove-Derived Fungus Penicillium citrinum
Source: Mar Drugs. 2016 Oct 10;14(10):177. doi: 10.3390/md14100177 (PMC5082325; doi:10.3390/md14100177)
Supplement: Supplementary file 1 [file marinedrugs-14-00177-s001.pdf]

Dihydroisocoumarins from the Mangrove-Derived Fungus *Penicillium citrinum*

Guo-Lei Huang, Xue-Ming Zhou, Meng Bai, Yu-Xin Liu, Yan-Lei Zhao, You-Ping Luo, Yan-Yan Niu, Cai-Juan Zheng and Guang-Ying Chen

List of Supporting Information

Figure S1. ^{1}H NMR (400 MHz, MeOD-d_4) spectrum of Compound **1** S2

Figure S2. ^{13}C NMR (100 MHz, MeOD-d_4) spectrum of Compound **1** S2

Figure S3. HMQC (MeOD-d_4) spectrum of Compound **1** S3

Figure S4. ^{1}H–^{1}H COSY (MeOD-d_4) spectrum of Compound **1** S3

Figure S5. HMBC spectrum (MeOD-d_4) of compound **1** S4

Figure S6. HRESIMS spectrum of Compound **1** S4

Figure S7. ^{1}H NMR (400 MHz, MeOD-d_4) spectrum of Compound **2** S5

Figure S8. ^{13}C NMR (100 MHz, MeOD-d_4) spectrum of Compound **2** S5

Figure S9. HMQC (MeOD-d_4) spectrum of Compound **2** S6

Figure S10. ^{1}H–^{1}H COSY (MeOD-d_4) spectrum of Compound **2** S6

Figure S11. HMBC (MeOD-d_4) spectrum of Compound **2** S7

Figure S12. HRESIMS spectrum of Compound **2** S7

Figure S13. ^{1}H NMR (400 MHz, $CDCl_3$) spectrum of Compound **3** S8

Figure S14. ^{13}C NMR (100 MHz, $CDCl_3$) spectrum of Compound **3** S8

Figure S15. HMQC ($CDCl_3$) spectrum of Compound **3** S9

Figure S16. ^{1}H–^{1}H COSY ($CDCl_3$) spectrum of Compound **3** S9

Figure S17. HMBC ($CDCl_3$) spectrum of Compound **3** .... S**Error! Bookmark not defined.**0

Figure S18. HRESIMS spectrum of Compound **3** .... S**Error! Bookmark not defined.**

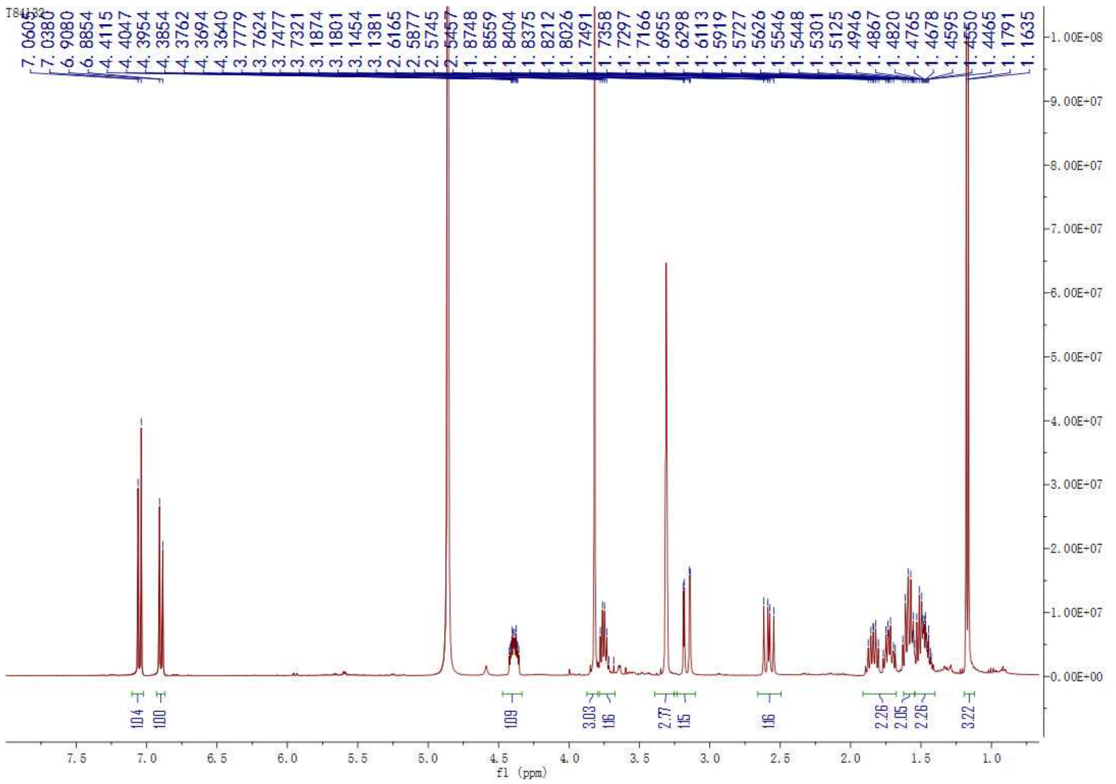

Figure S1. ^{1}H NMR (400 MHz, MeOD-d_4) spectrum of Compound **1.**

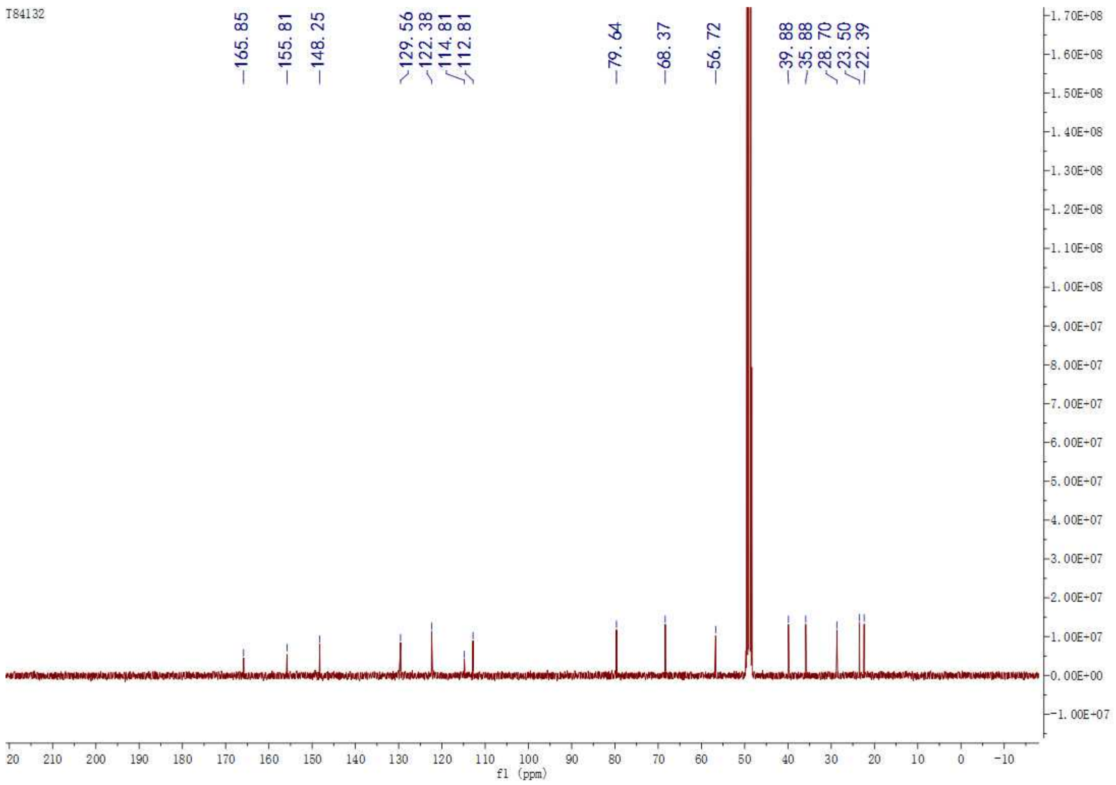

Figure S2. ^{13}C NMR (100 MHz, MeOD-d4) spectrum of Compound **1.**

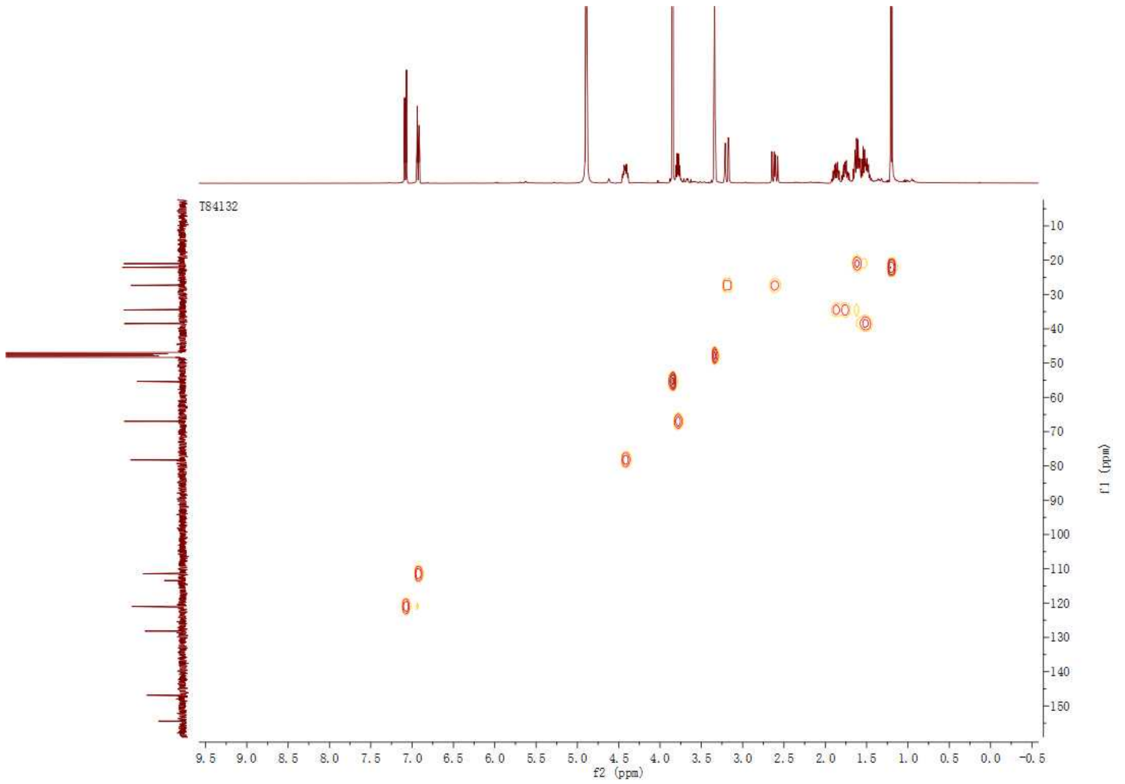

Figure S3. HMQC (MeOD-*d*4) spectrum of Compound **1.**

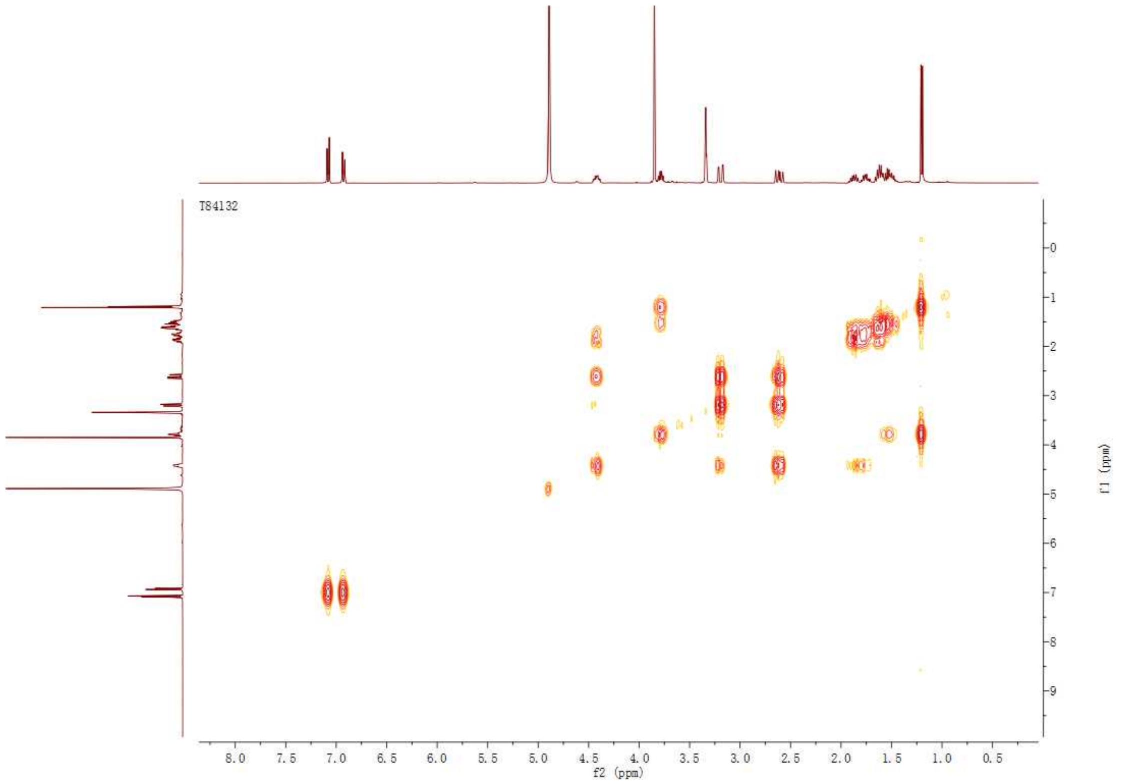

Figure S4. ^{1}H–^{1}H COSY (MeOD-*d*4) spectrum of Compound **1.**

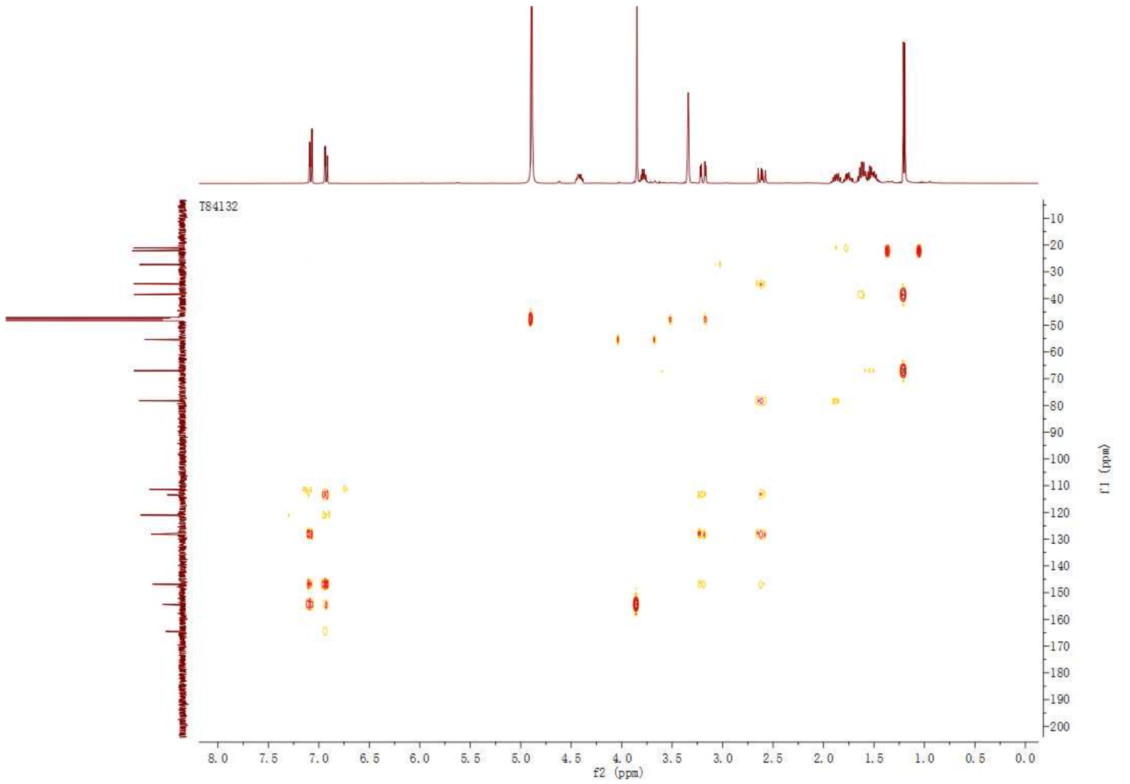

Figure S5. HMBC spectrum (MeOD-d_4) of Compound **1.**

T84 -132: HRMS (ESI) m/z calcd for $C_{15}H_{21}O_5^+$ $(M+H)^+$ 281.13835, found 281.13840.

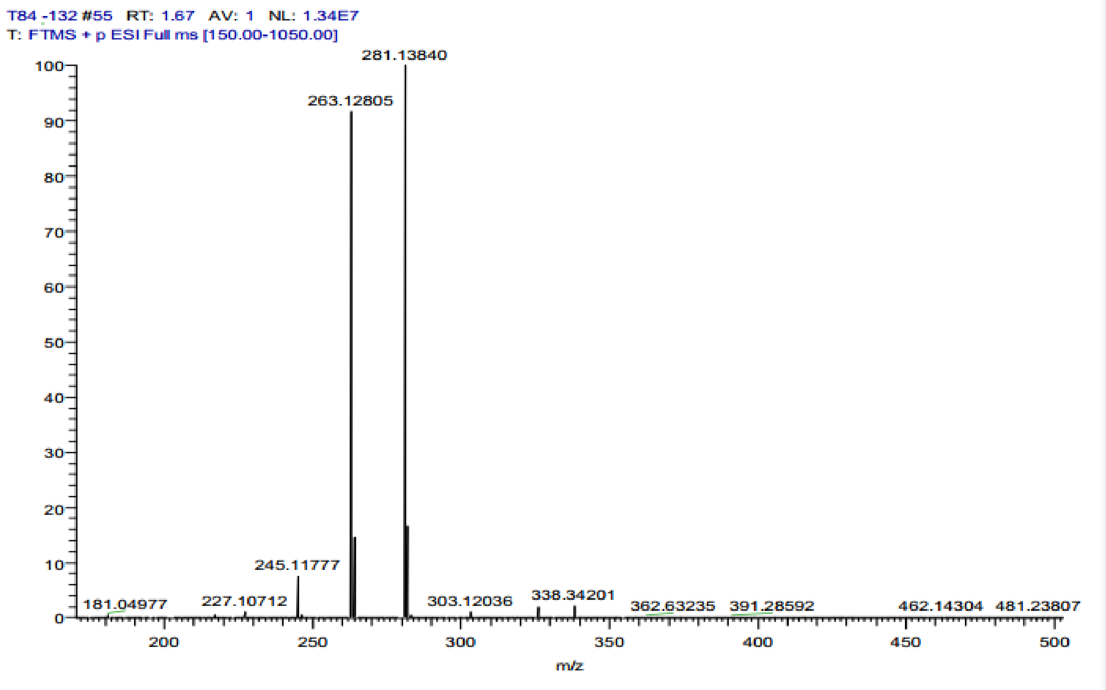

Figure S6. HRESIMS spectrum of Compound **1.**

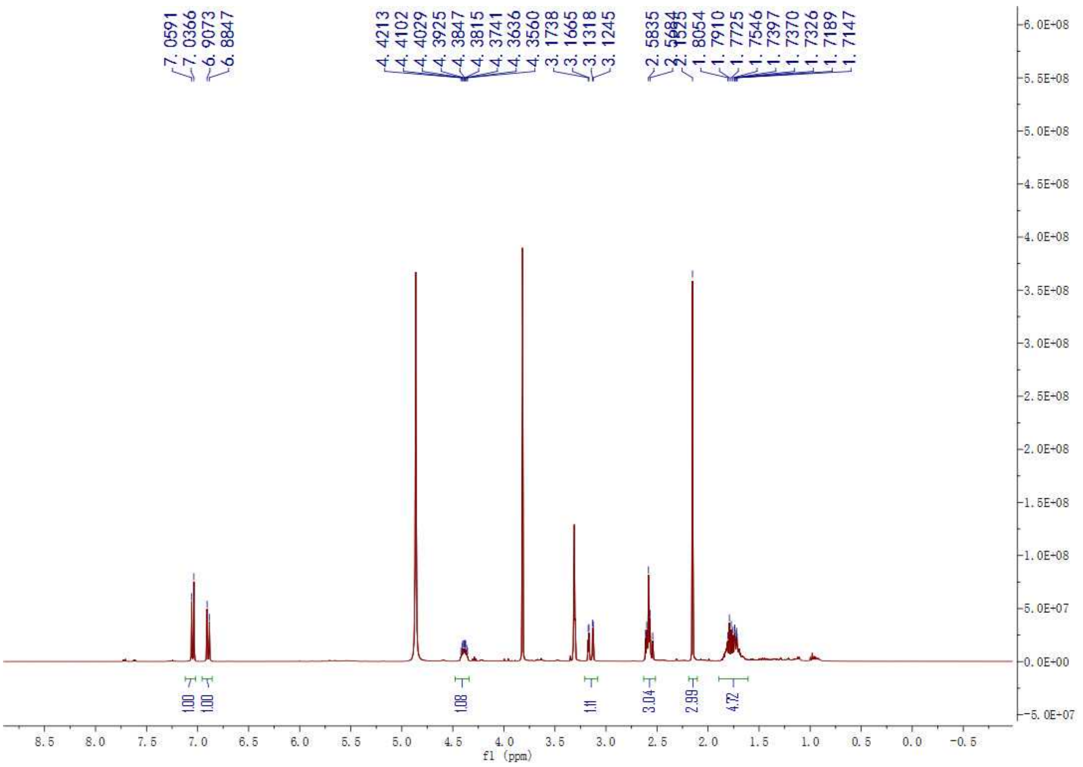

Figure S7. ^{1}H NMR (400 MHz, MeOD-d_4) spectrum of Compound **2.**

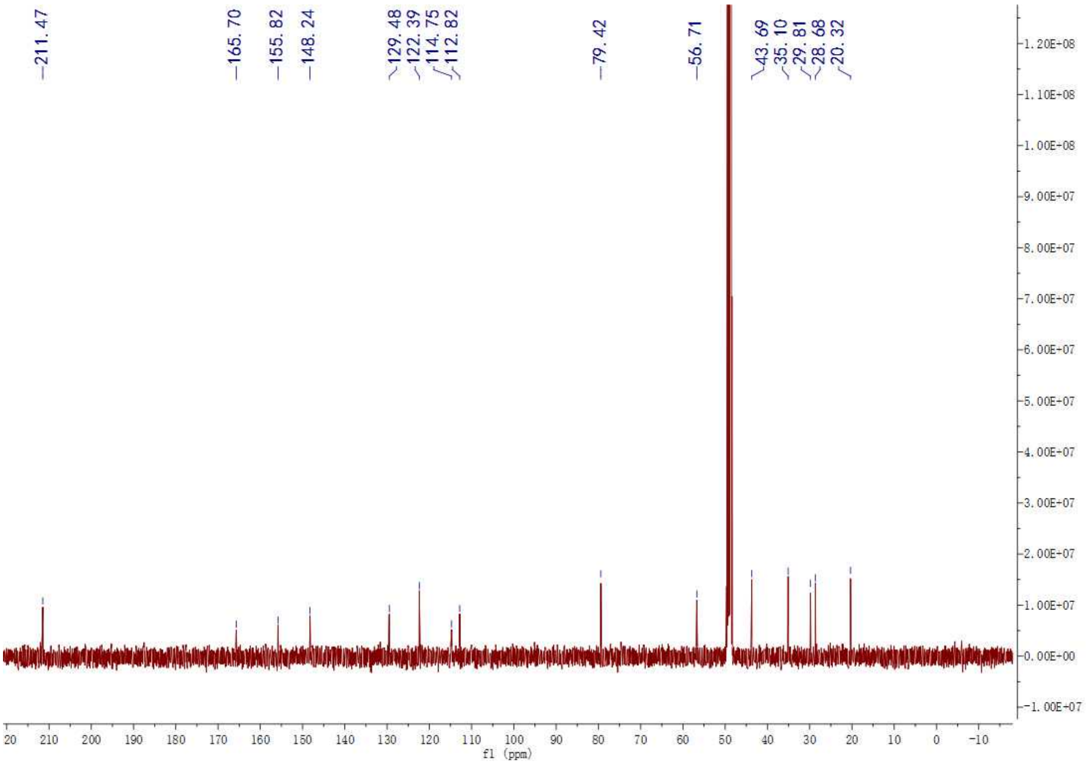

Figure S8. ^{13}C NMR (100 MHz, MeOD-d_4) spectrum of Compound **2.**

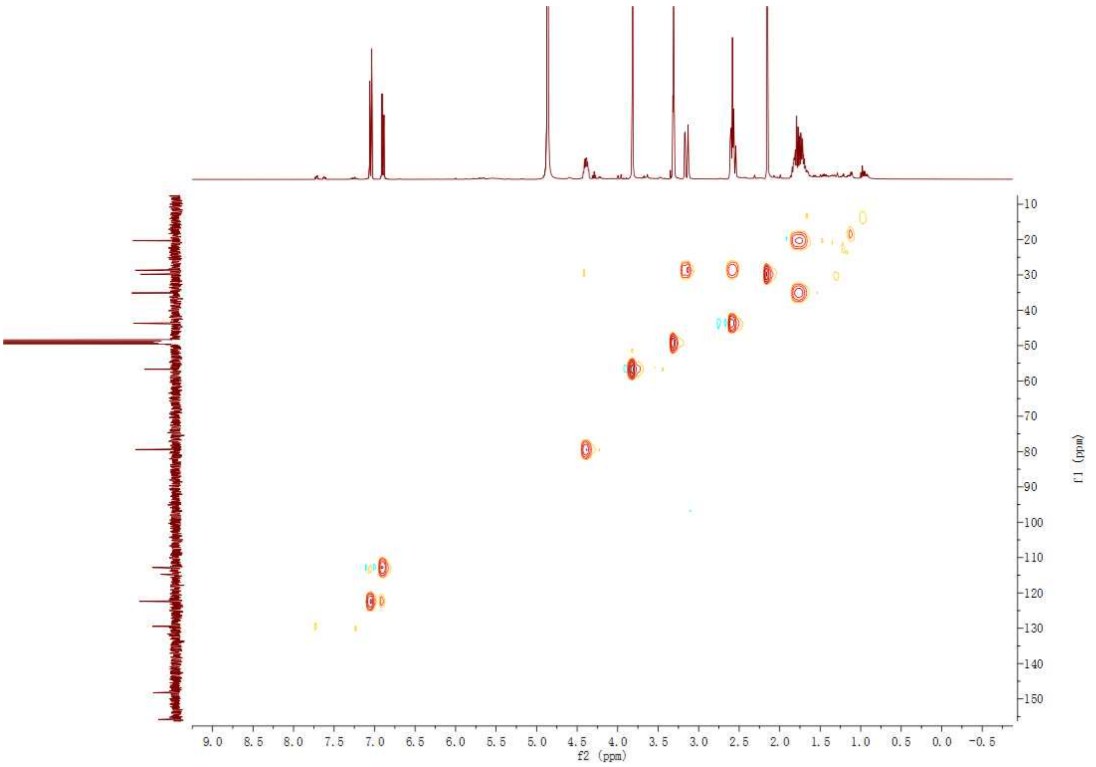

Figure S9. HMQC (MeOD-*d*4) spectrum of Compound **2.**

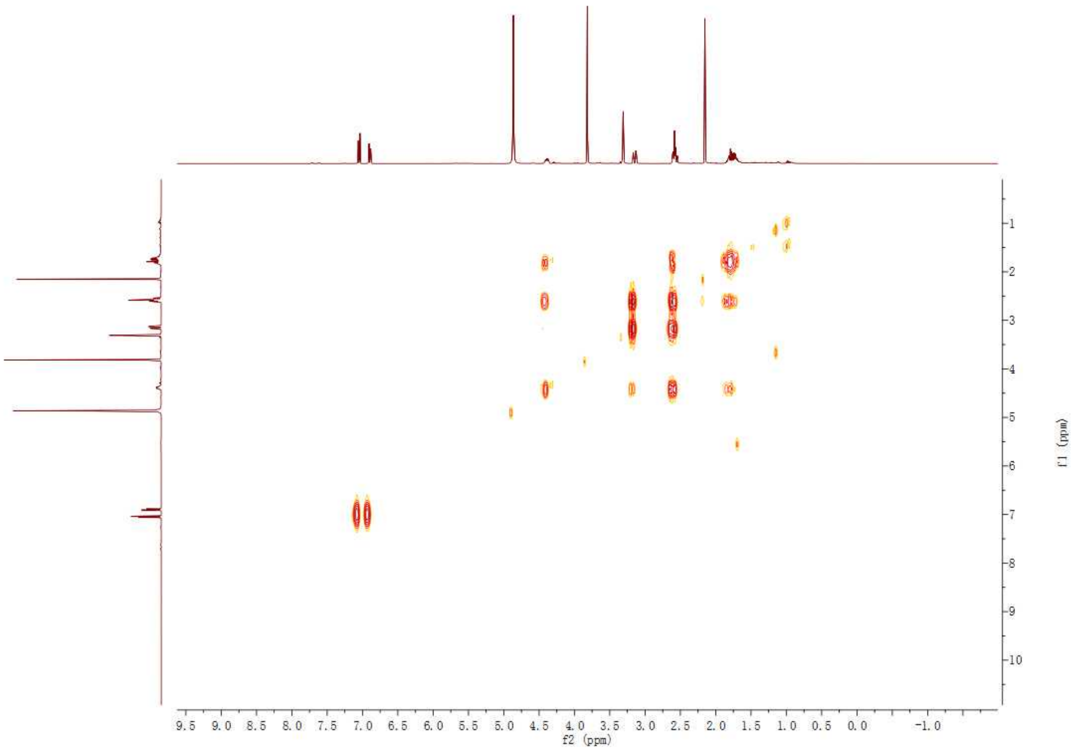

Figure S10. ^{1}H–^{1}H COSY (MeOD-*d*4) spectrum of Compound **2.**

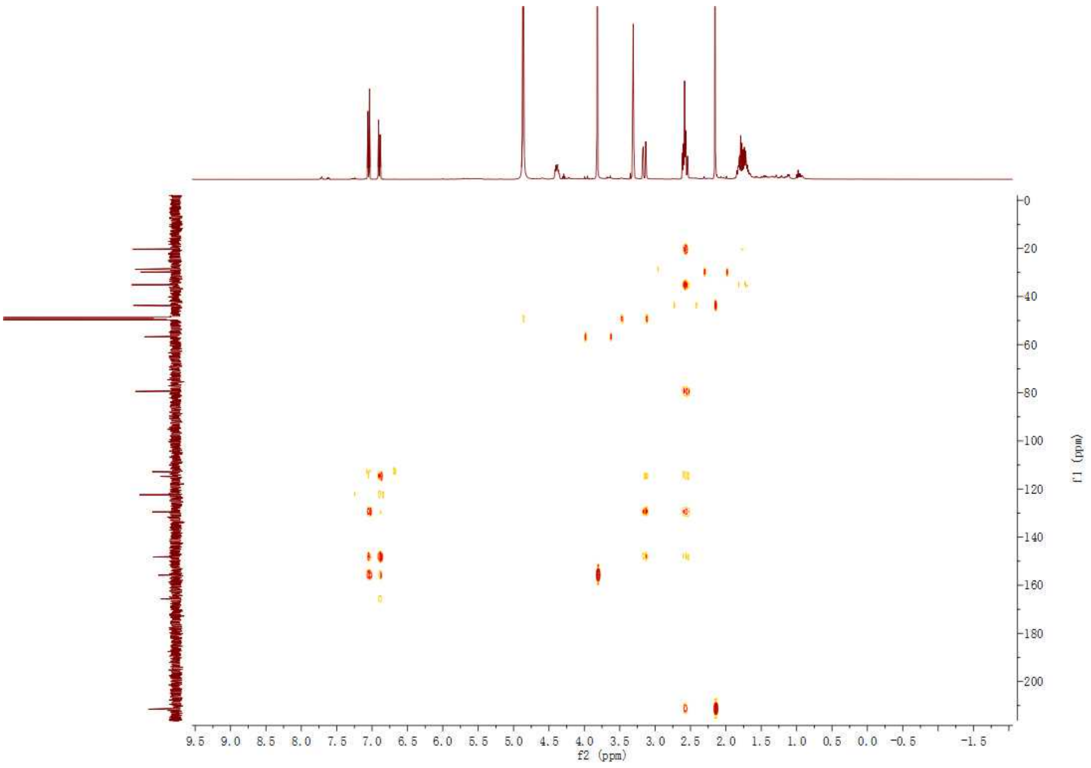

Figure S11. HMBC (MeOD-d_4) spectrum of Compound **2.**

T84 -121: HRMS (ESI) m/z calcd for $C_{30}H_{36}O_{10}Na^{+}$ $(2M+Na)^{+}$ 579.22007, found 579.22003.

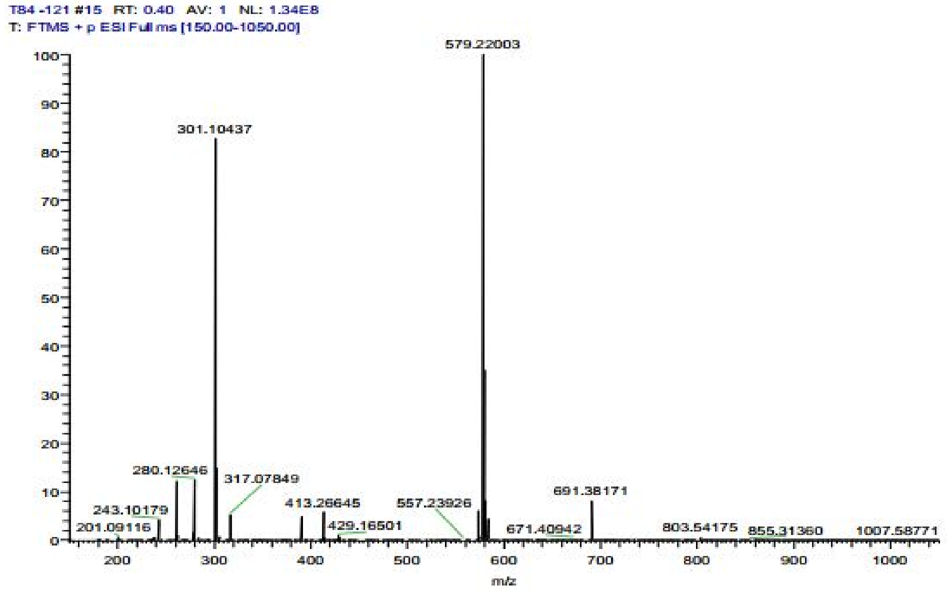

Figure S12. HRESIMS spectrum of Compound **2.**

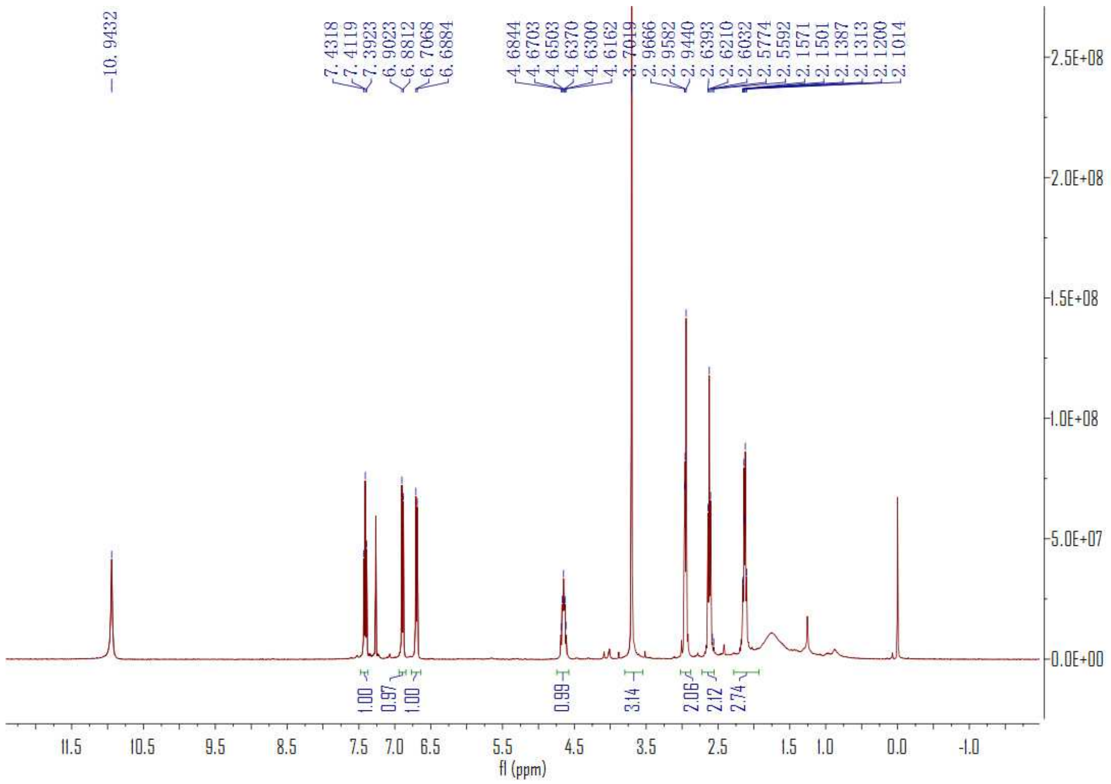

Figure S13. ^{1}H NMR (400 MHz, $CDCl_3$) spectrum of Compound **3.**

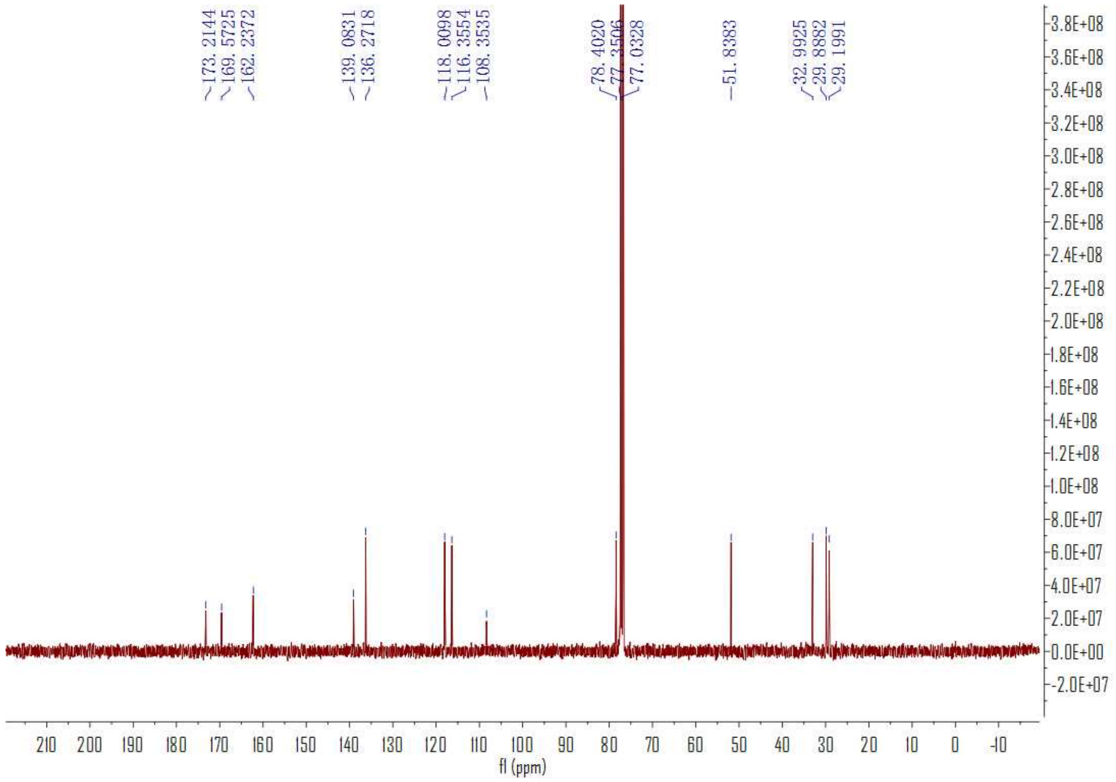

Figure S14. ^{13}C NMR (100 MHz, $CDCl_3$) spectrum of Compound **3.**

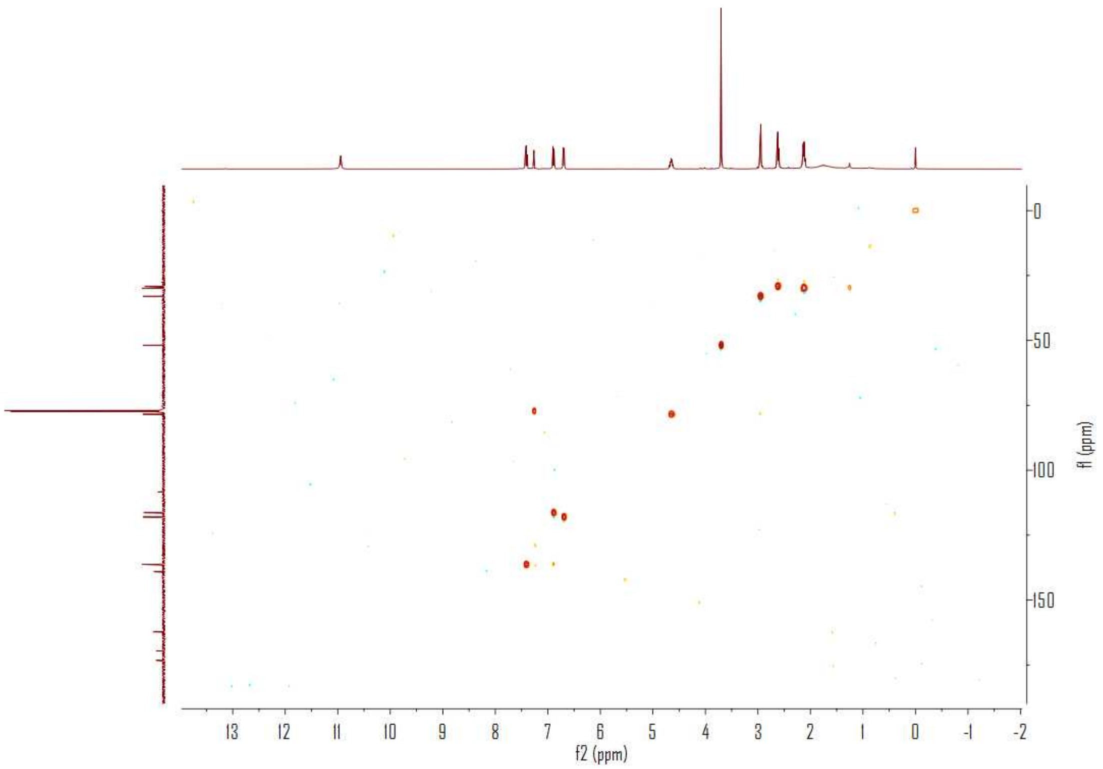

Figure S15. HMQC ($CDCl_3$) spectrum of Compound **3.**

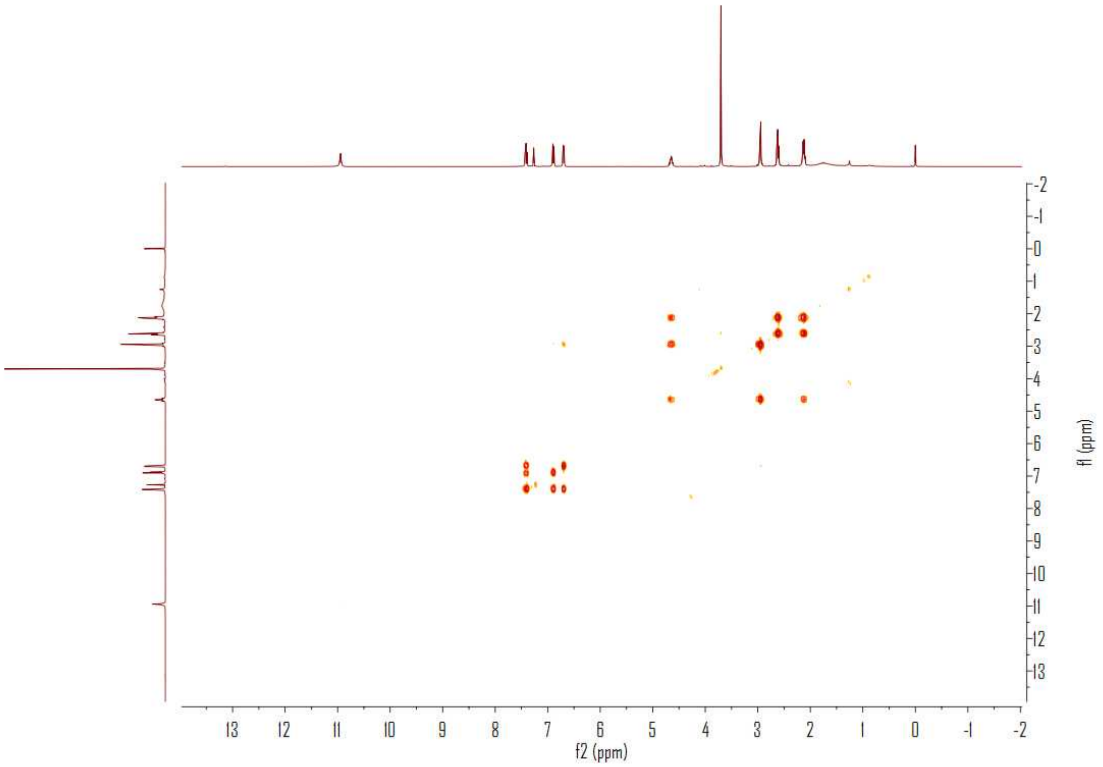

Figure S16. ^1H–^1H COSY ($CDCl_3$) spectrum of Compound **3.**

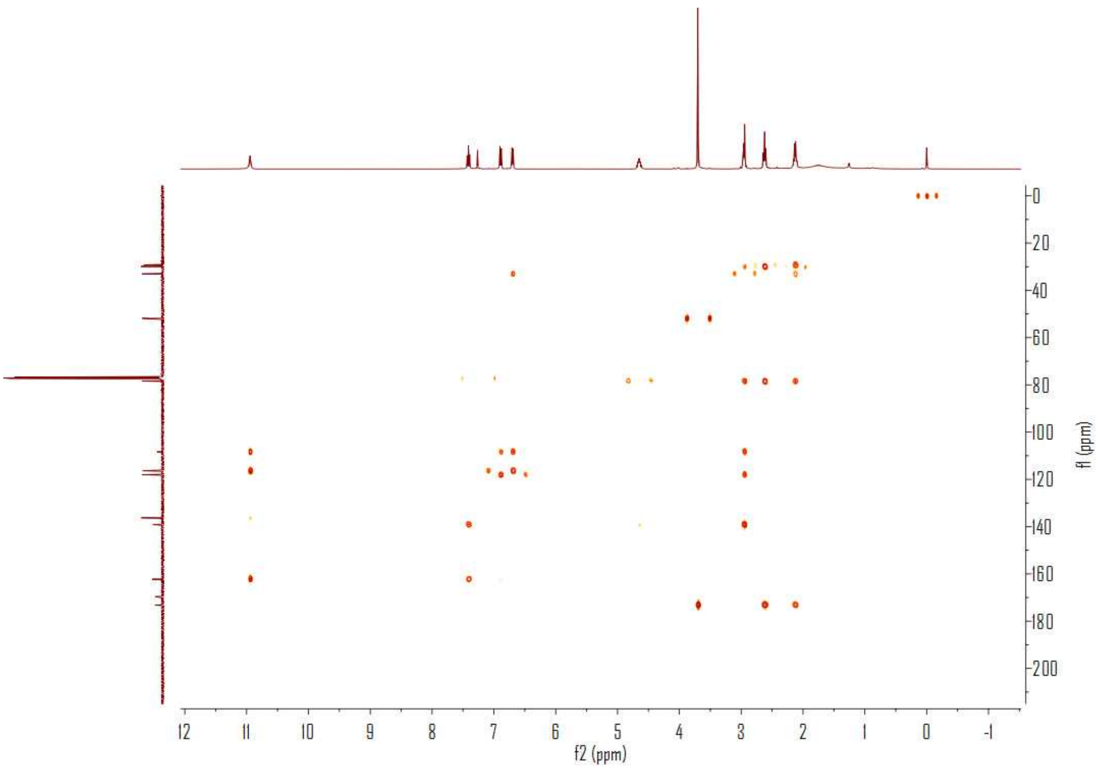

Figure S17. HMBC ($CDCl_3$) spectrum of Compound **3.**

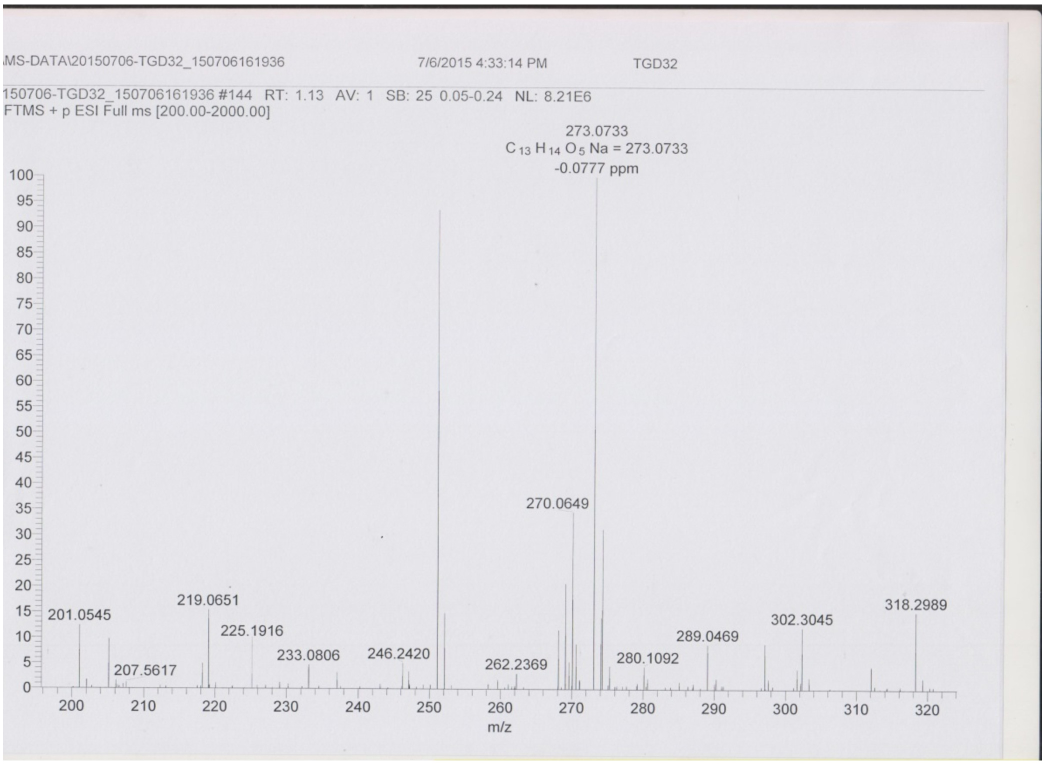

Figure S18. HRESIMS spectrum of Compound **3.**
